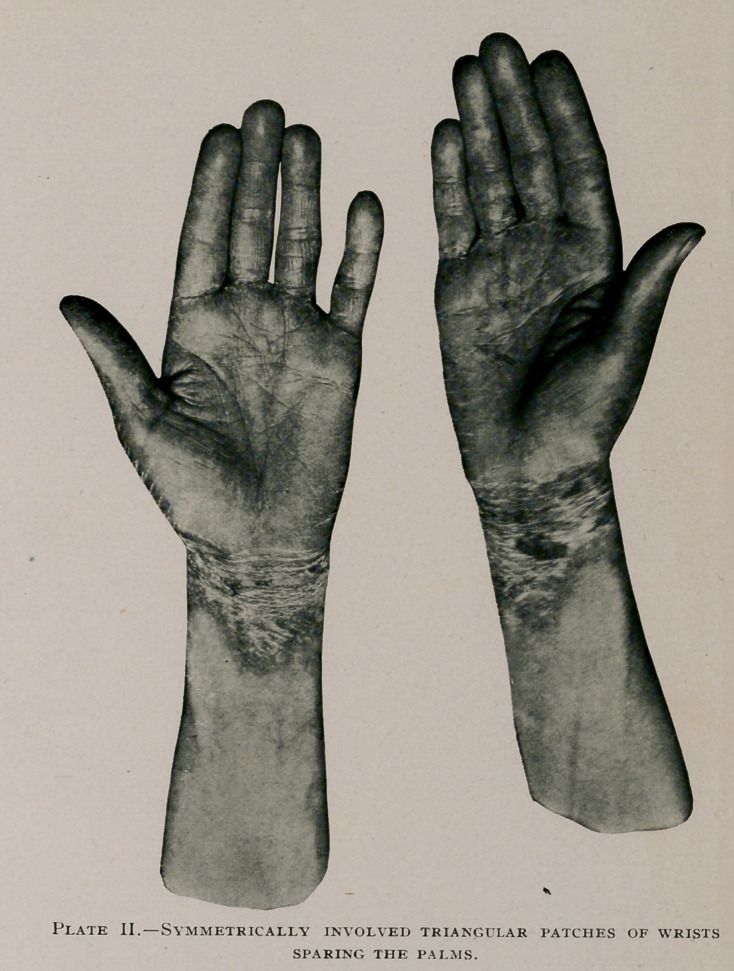# Pellagra in Buffalo

**Published:** 1911-05

**Authors:** Grover W. Wende

**Affiliations:** Buffalo, N. Y.


// BUFFALO MEDICAL JOURNAL

VOL. LXVI. MAY, 1911. NO. 10

### ORIGINAL COMMUNICATIONS

### Pellagra in Buffalo

BY GROVER W. WENDE, M.D.
Buffalo, N. Y.

THE patient, Mrs. E., housewife, age 33, was in care of her family physician, Dr. Jacob Goldberg, throughout her entire illness and, by his direction, was referred to me for the treatment of an eruption which made its appearance after she had spent portions of July and August in the country near Buffalo, where she was subjected to undue sun exposure.

*Family History.*—The patient's father was born in Germany and emigrated to this country when quite young. His death was accidental, at the age of 54. The mother is 70 years of age, well and hearty. There were nine children in the family, of which the patient was the oldest. The children spent their lives at home and were never jeopardized by travel. They were healthy, well-fed and clothed. In this family there have been no other cases of this nature.

*Past Personal History.*—The patient had measles when a child. During girlhood she was subject to no ailments so far as she can remember. Catamenia occurred at seventeen, and the attacks were regular. She was married at the age of twenty-three, bore two children and, at the birth of the last, suffered laceration of the cervix. During her entire life she received good, nourishing food in plenty. Corn or maize never constituted a specialty in her diet. She had never traveled; the only time she was out of the county being a period of eleven days, during her wedding-trip, when the objective point was New York City.

*History of Disease.*—Four and one-half years ago, September, 1906, immediately after a trachelorrhaphy and seven months after the birth of her child, she became fretful, weak and nervous, gradually growing worse until the following spring (1907), when diarrhea set in for the first time alternating with constipation. This condition lasted throughout the summer and until cold weather began, when the diarrhea was lessened and her general condition was improved. During the summer of 1908, the diarrheal symptoms returned, with loss of appetite, cankered mouth, loss of interest in household affairs, unusual weakness, even the gait lacking normal positiveness, and an inclination for rest accentuated.

As cold weather again approached the diarrhea improved, nevertheless she continued irritable and mentally depressed, frequently throwing herself upon the bed without removing her garments. Upon the approach of the warm weather of 1909, the gastric symptoms were considerably exaggerated, attended by diarrhea that sometimes lasted a week, with as many as thirty watery stools a day, as usual followed by constipation, but not to the former degree. In the winter of 1909-10 there was another improvement in respect to the gastro-intestinal symptoms, but an increase in the nervous irritability. At this time a marked change occurred in her catamenia which became very irregular and excessive, lasting for seven or eight days. For the first time during cold weather periodical attacks of diarrhoea occurred, the patient became weaker and was obliged to remain in bed most of the time; her gait was so seriously affected as to simulate paralysis. Her feet became uncontrollable and acted like club feet, with limping, pain and fatigue. In April, 1910, upon advice, she procured a brace which enabled her to walk without much difficulty. Upon the return of summer, her diarrheal symptoms re-appeared somewhat aggravated; she complained of more pronounced burning sensations in the mouth and developed a markedly capricious appetite for various kinds of food, especially ice cream. An illness of her child which necessitated constant care, worry and loss of sleep, caused the mental depression to be constant: there were symptoms of melancholia, ending in hallucinations and delusions —she claiming that she had no friends and that even her husband and brother were against her; this condition became so aggravated that on May 4, 1910, it became necessary to have her examined in lunacy and she was pronounced insane, but was not committed to an asylum, being cared for at home; after ten days rest under morphine her mania cleared up.

The attempt to obtain any definite history relative to skin manifestations was unsuccessful, although the patient stated that during the last two summers, the skin of the face became darker, and contained spots which she attributed to an unusual sensitiveness to the sun. She did not remember that the hands were involved, but, in July, 1910, just before leaving for the country, she noticed that the finger-tips were reddened and very sensitive. Immediately after reaching her destination the skin affection rapidly spread to the fingers and finally covered the hands; the unusual exposure to the sun undoubtedly aggravated this symptom, the burning and itching becoming constant. The gastro-intestinal symptoms improved while she remained in the country.

The patient was under constant observation by Dr. Goldberg for more than four years and he considered her case one of neur-

PLATE I.—SYMMETRICAL LESIONS OF HANDS. SKIN DRY AND EXFOLIATING.

asthenia with general debility. Various tonics were continuously prescribed during this time.

*Present Condition.*—Upon her return to the city, August 30, 1910, the patient constantly moves her hands and she complains bitterly of her suffering. Solemnity and sadness are both clearly expressed by her countenance and manner. The face shows unusual lifelessness, as though she had been suffering intensely; if forced she laughs in a silly way; when questioned, her answers lack definiteness. She is evidently poorly nourished. The skin of her face is of a dark color covered with irregular, or, rounded, fawn-colored patches. A physical examination of her heart reveals weakness; of her lungs, shows them to be normal. The abdomen is greatly distended; the liver and spleen are not palpable; the knee reflexes are greatly exaggerated. The pupils appear somewhat dilated, the lips are dark colored, fissured, bleeding and scaly. The entire buccal mucosa is denuded of epithelium, presenting a bright red appearance, with an occasional bleeding point; the gums are swollen and show apthous ulcers, as well as long, stringy pieces of epithelium; the tongue is bright red with enlarged papillae, as in scarlet fever; the breath is foul and the whole condition of the mouth resembles an advanced stage of mercurial stomatitis. The skin of the body is dry and rough, the hair lustreless. The hands, as shown in photographs, present the most pronounced evidence of disease, the entire dorsum of both hands being symmetrically covered by single triangular patches with apices extending up the wrist toward the radial side and fading into unaffected surfaces of normal skin; the whole of the invaded surface presents a puffiness and a brownish red color which does not disappear under pressure; the redness appears as if associated with deep-seated hemorrhages; the surface is covered with scaly, exfoliating skin; over the phalanges and the interdigital spaces a chocolate color is diffused, deeper than that manifested elsewhere. The palmar surfaces of both hands are unaffected. The flexor surfaces of the wrists present similar conditions to those of the dorsal except that the apices of the symmetrical involved areas extend toward the ulnar side fading as before into healthy skin.

*Subsequent History.*—August 18, 1910. The patient, having now been confined to her house for a fortnight and no longer subjected to the damaging sun rays, the acute inflammatory symptoms have disappeared from the skin. After desquamation there is more bluish-red pigmentation that does not disappear under pressure; the use of a simple soothing ointment was probably a factor in producing this result. She continuously complains of burning in the stomach, nausea, excessive thirst, loss of appetite and a longing for ice cream. She is utterly prostrated. The facial expression is distressing. Temperature, 100° F.; pulse, 110; respiration, 22.

Blood examination made by Dr. Nelson G. Russell, shows—haemoglobin, 78 per cent.; reds, 4,066,666; whites, 8,200; polynuclear, 68 per cent.; small mononuclears, 12 per cent.; large mononuclears, 7 per cent.; transitional, 10 per cent.; eosinophiles, 1 per cent.; eobasophiles, 2 per cent. (both red and blue granules with lightish-blue polynucleus). Red cells, good color; considerable variation in size, some quite large ,some quite small. Platelets normal.

Urinalysis shows:—Specific gravity, 1,025; yellow color, and foul odor; albumen present; the sediment shows a great many pus cells and many granular casts.

Examination of feces shows a chocolate colored material, separating into two layers, the upper a fluid and the lower a semifluid rather coarse, granular material. Microscopical examination reveals presence of blood; otherwise nothing unusual.

For the next two weeks the temperature ranged from 98° F. to 101° F. The patient would respond to emphatic questions but the responses would usually be followed by irrelevant questions or comments relating to all sorts of subjects. Mental excitement and physical restlessness were very marked symptoms. This condition continued in varying degrees until the 20th of September after which the temperature became subnormal; there was a marked accentuation of all the symptoms and the presence of complete prostration. The diarrhoea was now almost continuous. There was extreme tenderness over the entire body, especially along the spine.

During the last two weeks the prostration became intense, and the mental condition markedly aggravated; temperature subnormal, ranging from 96° F. to 97.5°F. both by mouth and by rectum. Diarrhoea marked, from seventeen to twenty stools a day, little influenced by astringents. The cardiac centers and not the respiratory centers seemed to be involved; two days before death the heart sounds could scarcely be heard; coma supervened and death came Monday, October 10, 1910. No autopsy was permitted.

*Summary.*—The clinical features of this case correspond almost exactly in detail with those of pellagra, particularly those referred to the alimentary tract, to the cerebro-spinal system, and to the skin. Some difficulty would naturally arise in making the diagnosis at an early period as nothing connected with her life or medical history throws any light upon the origin of the disease so strangely affecting her. This woman apparently enjoyed good health until the spring following the birth of her last child, when she became much fatigued from undue exertion and had sensations of weakness especially in the lower extremities, accompanied with pain in the epigastrium, and diarrhoea of short duration followed by constipation. Later there is a history of stomatitis associated with mental depression and gastric disturbances. As the disease progresses these symptoms appear to grow worse, until they culminate in an attack of acute mania of short duration, followed by great depression. One rather interesting feature is the early onset of weakness of the extremities, soon associated with neuritis, causing disturbed gait and finally simulating paralysis.

All these are symptoms corresponding to those of the various stages of pellagra. Notwithstanding this, there would never have been any suspicion as to the nature of this disease without the cutaneous manifestations which though in themselves rather harmless are, however, of great diagnostic importance. The symmetrical, even, triangular distribution with apices spreading centrally over the wrists, on the dorsum toward the radius, on the palmar surfaces toward the ulna, while the palms are spared is almost pathognomonic. While the color of the eruption was not characteristic during its continuation, yet this, at any one time, was sufficiently so to be distinguishable from any other dermatosis and this showed a bright red at first, disappearing under pressure to a dull red; later with the addition of pigment, to a reddish-brown, and finally chocolate, uninfluenced by pressure. The chloasma on the face that had existed for a number of years may have been due to the systemic conditions as is observed in other constitutional diseases. The chief reason for directing attention to the dermatological symptomatology is because of its importance in the establishment of a diagnosis. Errors have even been made by experts all over the country, because they have failed to note the relations of the skin changes which should early have aroused suspicion. As far as the literature has been searched, this is the first case of pellagra appearing in a person born in Buffalo, and probably the second reported as native in New York State.

*Pellagra in New York State.*—Imported cases were first recognised in this state as far back as 1864. Dr. John Gray (*Am. Jour. Insan.*, 1864-1865, Vol. XXI, pp. 223-227) of the Utica State Insane Asylum, reports a case of pellagra in a foreigner coming under his observation, who died at the institution. Subsequently, Samuel Sherwell (*Jour. Cutan. and Ven. Dis.*, 1882, No. 1, p. 142) reported a case of pellagra in an Italian sailor, age 35, who died in the hospital after being under observation for three months; he had lived before the mast in small and poorly outfitted Italian brigs, furnishing his supplies from home, from a pellagra-infected district, maize constituting one of his foods. In 1902, Sherwell (*Trans. Am. Der. Assn.*, 1902, p. 76) reported another case in an Italian sailor, age 30, who had suffered from a series of attacks during a number of years, and who when coming under observation was considerably emaciated, and finally died of pellagra.

Dr. Howard Fox (*N. Y. Med. Jour.*, Feb. 26, 1910) reports a case of pellagra presented by him before the Academy of Medicine, which was imported from Blue Ridge, Georgia, for the purpose of calling attention to the disease on account of the serious aspect of its appearance in this country. In the same paper, Dr. Fox refers to another undoubted case seen by Dr. John A. Fordyce, during his service at the City Hospital; this patient was an Italian woman, with mental depression and a dermatitis on the hands and arms. He also mentions that Dr. Claude Lavinder lately saw a case at the Marine Hospital, Staten Island.

Dr. A. Caccini (*Med. Record*, March 11, 1911, p. 428 ) reports eight cases. All of these with the exception of one were in males. One patient was born in New York and lived there during his entire life. This is probably the first case recorded, appearing in a person born in New York City, or the first indigenous to the State of New York.

*Pellagra in the United States.*—It is a difficult task to form even an approximate estimate of the number of cases of pellagra that have existed in this country. The credit of first collecting statistics as to number and location of cases of pellagra in the United States, is due a National conference under the auspices of the South Carolina State Board of Health, held at Columbia, in 1908-1909, under the presidency of Dr. J. W. Babcock. Most of the papers there presented were published in the *Journal of the South Carolina Medical Association*, Vol. IV, 1908, and Vol. V, 1909, and have been collected in pamphlet form. Five to seven thousand cases have occurred during the last five years. From the literature it may be approximately determined that pellagra is endemic and relatively numerous in the states of Alabama, North and South Carolina, Florida, Georgia, Illinois, Louisiana, Mississippi, Texas, Tennessee and Virginia; endemic and relatively few in Arkansas, California, Kansas, Kentucky, Maryland, Oklahoma, and Pennsylvania; sporadic and imported in Indiana, Iowa, Michigan, Missouri, Massachusetts, New Mexico, New Jersey, Ohio, Rhode Island, Vermont, West Virginia, Wisconsin, and Washington.

Pellagra has long been believed to be caused by the consumption of damaged maize. This theory is almost as old as the dis-

PLATE II.—SYMMETRICALLY INVOLVED TRIANGULAR PATCHES OF WRISTS SPARING THE PALMS.

ease itself and was at one time quite universally accepted, but recent arguments against it are both numerous and cogent. It would be almost impossible to set forth the arguments in favor of the "zeists," or, the enormous amount of facts strongly in favor of the "antizeists." Attempts have been made to establish the origin of pellagra in the United States in corn-eating territories, and it is well to give forth the following arguments that point in that direction.

A paper on the etiology of pellagra, by J. J. Watson (*Jour. So. Carolina Med. Assn.*, Nov., 1908) gives the main facts of Lombroso's theory of the connection between maize and pellagra which is quite universally accepted. In moisture certain fungi (Penicilium, Aspergillus, and the like) grow upon maize and produce a toxin which is the cause of pellagra. Lombroso claimed that his experiments upon men, dogs, and chickens proved his theory, and Watson himself is thoroughly convinced that damaged corn is the cause of the disease in the United States. While the local corn is thoroughly cured, he believes that western corn becomes heated before reaching its destination, and when sold is infected by fungi, as he has demonstrated from samples secured from the markets.

D. R. Silver (*Jour. Am. Med. Assn.*, Feb. 5, 1910) on the subject of corn and pellagra, publishes a letter which he had received from the ex-president of the Ohio Grain Dealers' Association which is very suggestive in the light of the spread of pellagra in the United States. The latter says that he never had trouble with corn shipments until the last six years, during which time many farmers made no effort to cure the corn sufficiently to bear transportation; therefore, before it reached its destination it became heated. If the corn becomes too bad the consignee refuses to accept it, in which case it is put through a dryer; as fast as it is dried and cooled it is disposed of; this was sold at a discount of 10 to 50 per cent. (but not for distillers' use, as it will not make spirits); some of it is exported after mixing with good corn. The best is selected and made into meal which costing so much less, can be sold at an attractive price.

Dr. Silver believes that this inference is justified from the fact that the very time during which the letter-writer had the most trouble with spoiled corn coincides with the rapid spread of pellagra in the South. He also mentions Buffalo as one of the cities which has extensive dryers and large mills.

Being somewhat interested as to the final disposition of the corn sent to Buffalo, I find that during last year (1910) alone, Buffalo handled twenty million bushels of corn which came via the lakes. Upon its arrival, it had often been overheated in the vessels, a natural tendency of such masses of improperly cured grain. After unloading, it was handled several times, cooled and dried by a fan system until the final shipment was made. Most of this corn found its way to the New England market for consumption. Some of it was exported. The corn that was damaged by water and not accepted by the consignee, was sold to local dealers who kiln-dried, ground and made chicken-feed of it. The amount of damaged corn handled in this manner is comparatively small, especially during the period of the last six or seven years. It is an interesting fact to note, that in the states in which most of the corn that came by the way of Buffalo is sold, there have been but three sporadic or doubtful cases of pellagra reported. Naturally, the states in which the disease has appeared endemically, or in unusual numbers, receive shipments from the same sources as this port, and there is no reason why the effects of the final consumption of this corn in the Eastern states should be different from that consumed in the South.

At the St. Louis session of the American Medical Association, before the section on Dermatology (*Jour. Am. Med. Assn.*, July 2, 1910, p. 65) a number of pellagra cases were presented by Drs. George A. Zeller and R. P. Price, a general discussion by a great many recognized authorities following. It is well to consider the great educational value of the bringing of these patients a long distance to give the general profession an opportunity to see this disease; no less than four thousand physicians saw these patients and listened to the experiences of Dr. Zeller and others.

Physicians returning home after attending this meeting discovered cases in sections of the country where heretofore the disease had not been recognized. In the discussion which followed, attention was directed to the fact, that at last this country is awakening to a consideration of this disease, with the expectation that some definite action will soon be taken on the grave problems presented by the existence of pellagra in so many sections of the country.

During the discussion of the symptomatology presented by the above cases, attention was called to the fact that the then shown skin lesions were not characteristic and that it would be difficult to recognise the disease by these skin manifestations which are never very pronounced during the early summer, but during the latter part of the summer the skin through the influence of the sun displays its greatest reaction and presents pathognomonic lesions; although the persons presented were from insane asylums it was brought out that a great many cases occurred in private practice, and not necessarily among the poorer classes.

The importance of pellagra to the alienist was further brought out in the variety of mental symptoms shown by these patients, which are usually identified as those of melancholia, although they may manifest themselves in many forms, from simple neurasthenia to manic—depressive insanity, and may even simulate general paresis of the insane.

It was also shown that the disease is not communicable in the ordinary sense of that word, although there are instances in which several members of the same household were affected. In one institution, a Baptist orphanage, 17 cases developed, the disease being introduced by a child three years old, which child was a member of a family of four and all four developed the disease. Whether the mode of transmission is by a fly or other insect, or through food, is not known.

It was shown that pellagra is essentially a chronic condition; pellagrins may live from twenty to thirty years; that it occurs at all ages, having been observed in a child in arms, and in very old people. One of the striking features of the American type is its high mortality; in Alabama, in one asylum, the mortality was 68 per cent. Statistics based on asylum cases give a mortality of 67 per cent. of the patients; 10 per cent. become insane, and these rarely ever recover. In Southern Europe, in 1884 the mortality was 13 per cent. In 1905 it had been reduced to 4.3 per cent., and in 1907 it had been still further reduced to 0.7 per cent.

In considering the etiology of the disease some were not quite prepared to accept the damaged maize origin.

It was brought out that the microscopical findings in the stools of pellagrins consisted of muscle fibers, starch, crystals of various kinds, fats, oils, muco-pus, blood, vegetable cells and fibers, and parasites, such as amebas, flagellates, ascaris, uncinaria, trichuris, oxyuris, and the like. These findings are of interest in connection with the possible etiology of the disease, as some believe it is due to an intestinal toxin, and this view is strengthened by the fact that in the milder cases, those that were not too far advanced, the mental symptoms were relieved very markedly by free elimination through the intestinal tract.

471 Delaware Avenue.

---

A Case of Hook-Worm Infection Occurring In New York.—Harlow Brooks of New York (*Medical Record*), describes a case of hook-worm disease occurring in an Irish-American of New York, who had contracted it by working with a gang of Italian laborers. The patient had been treated for two years for pernicious anemia in several hospitals, without benefit. It was discovered that the stools contained hook-worm ova and parasites. After treatment with thymol, which was unsuccessful and had produced unpleasant symptoms, beta-naphthol was used, and a cure resulted slowly.

## Figures and Tables

**Plate I. f1:**
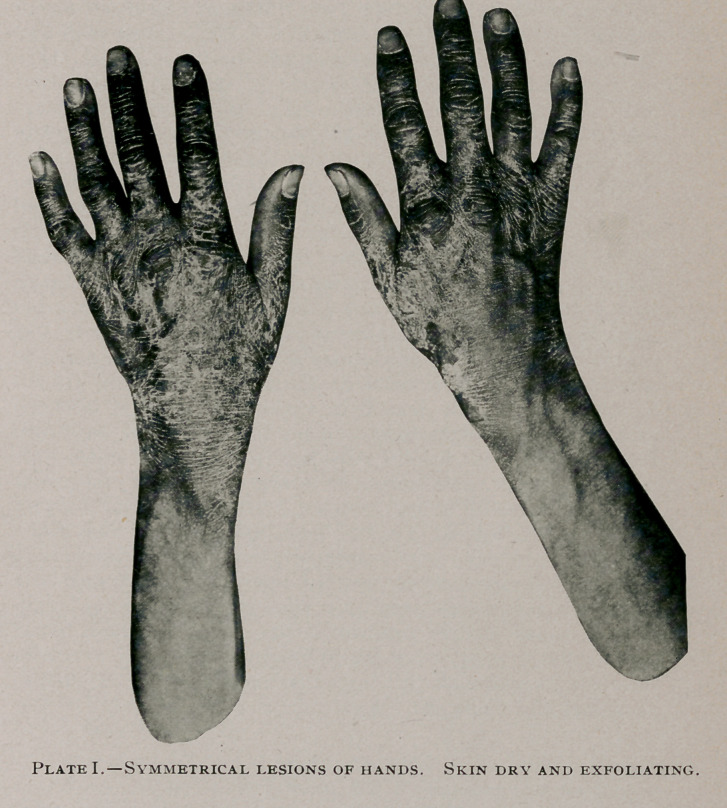


**Plate II. f2:**